# Supplementary figures and images for: A size-invariant bud-duration timer enables robustness in yeast cell size control
Source: PLoS One. 2018 Dec 21;13(12):e0209301. doi: 10.1371/journal.pone.0209301 (PMC6303054; doi:10.1371/journal.pone.0209301)

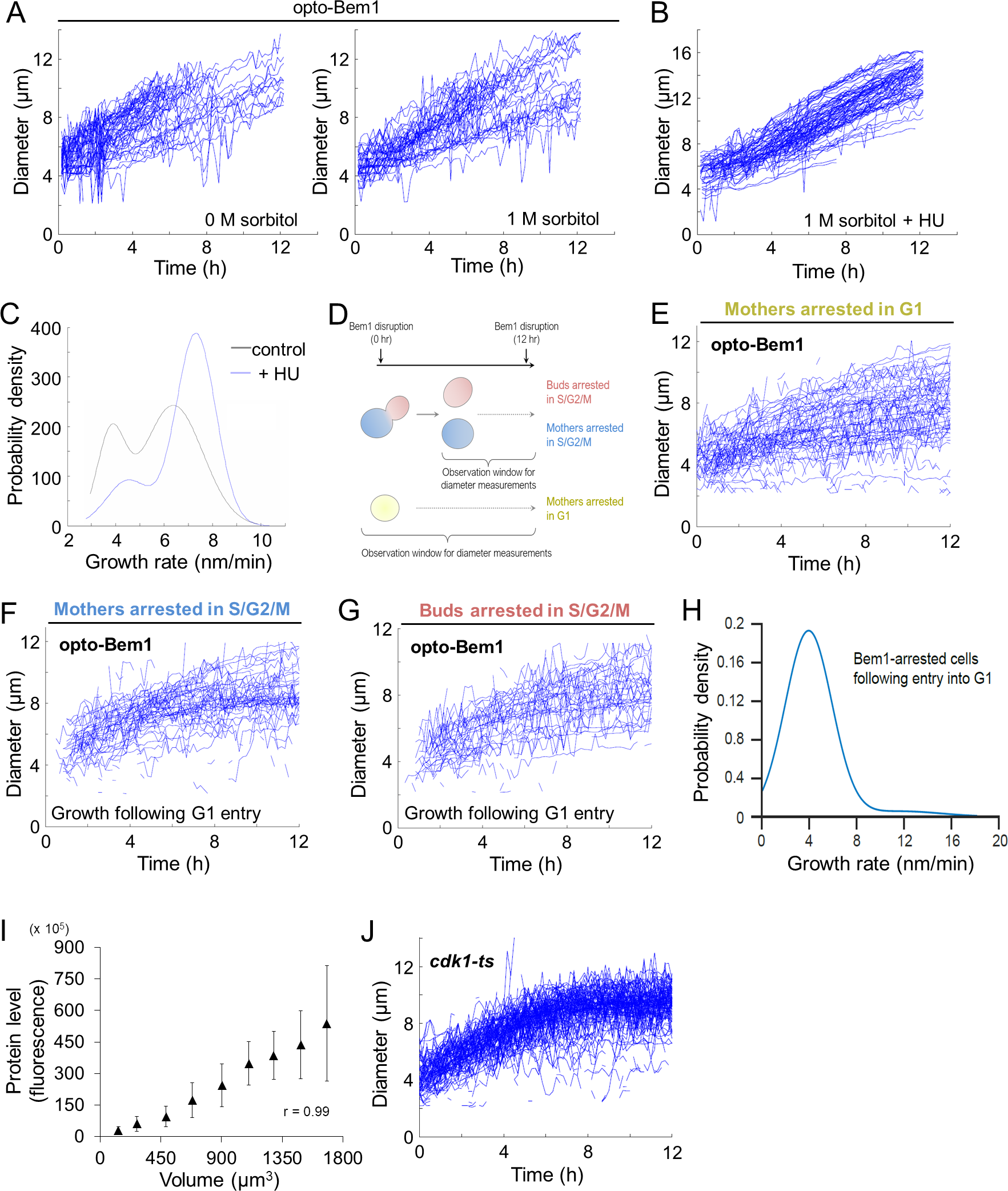

Supplement: S1 Fig — (A) Growth rates of single optoBem1 cells in synthetic complete media containing 0 M or 1 M sorbitol. (B) OptoBem1 cells were prepared as in Fig 2B and synchronized in S-phase via incubation with 0.2 M hydroxyurea for 3 h followed by washout using media containing 1 M sorbitol. (C) Probability distributions for growth rates measured in cells from S1A Fig, right panel and S1B Fig. (D-G) Growth rates of single optoBem1 cells in synthetic media with 1 M sorbitol. Asynchronous populations of cells were treated with red light illumination to disrupt Bem1 and cells were grouped based on initial cell cycle stage as depicted in (D): cells in G1 (E), mothers in S/G2/M (F), or buds in S/G2/M (G). Growth rates for cells not already in G1 (i.e., S1F and S1G Fig) were only measured following entry into G1, as indicated by these traces not beginning at ‘0 h’ and as depicted in S1D Fig. (H) Probability distribution for growth rates of Bem1-disrupted cells following entry into G1 (i.e., Fig E, F, and G in S1 Fig). (I) Fluorescence of exogenously-expressed PhyB-mCherry-Tom7 under control of an ADH1 promoter was measured in cells of indicated volumes. Cells were binned by mother volume in 200-μm increments. The average volume within each bin is plotted. N = 300 cells. Error bars, SD. r, Pearson’s correlation coefficient. (J) Growth rates of single cdk1-ts cells at 37°C. Cells were shifted from 25°C to 37°C 45 min prior to the start of the experiment to allow for Cdk1 disruption. (TIF) [file pone.0209301.s001.TIF]

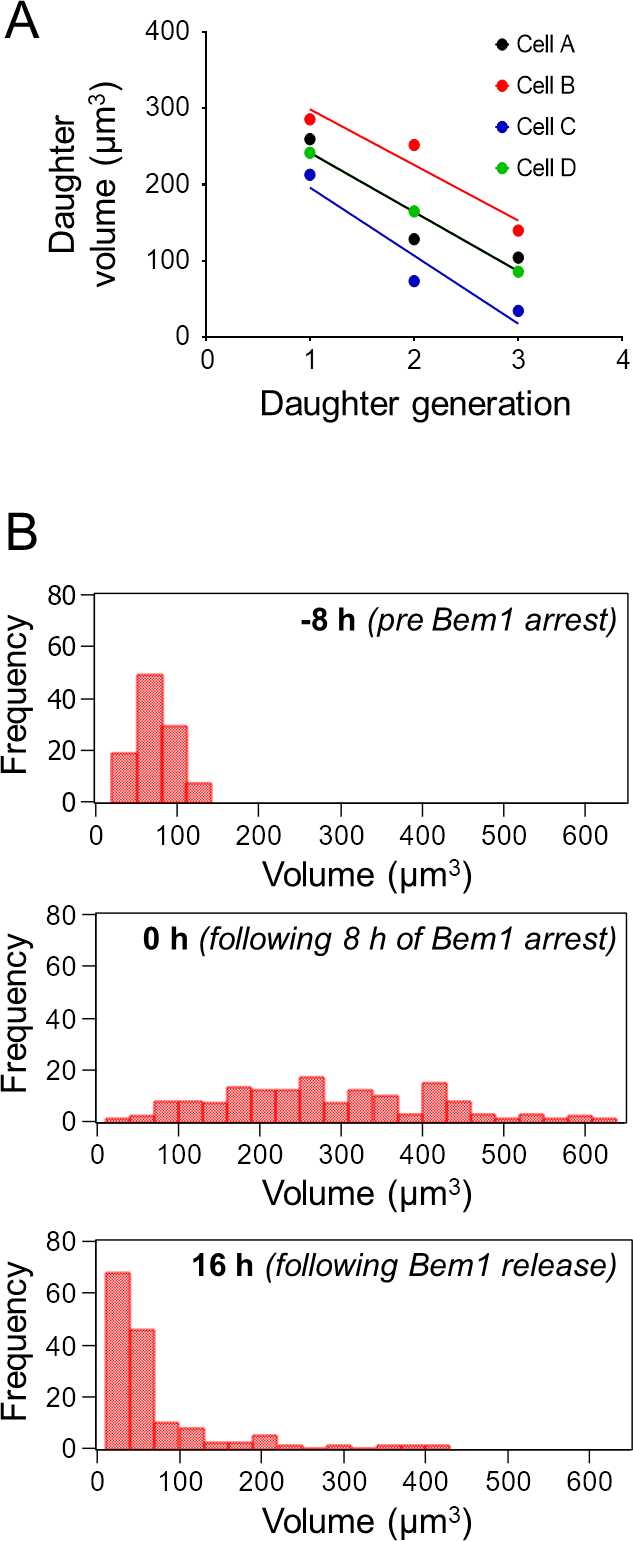

Supplement: S2 Fig — (A) Representative optoBem1 daughter cells from experiments in Fig 4C and 4D. Only the daughters of daughters were measured for each generation. (B) Histograms depicting cell volume distributions for indicated timepoints in Fig 3A. (TIF) [file pone.0209301.s002.TIF]

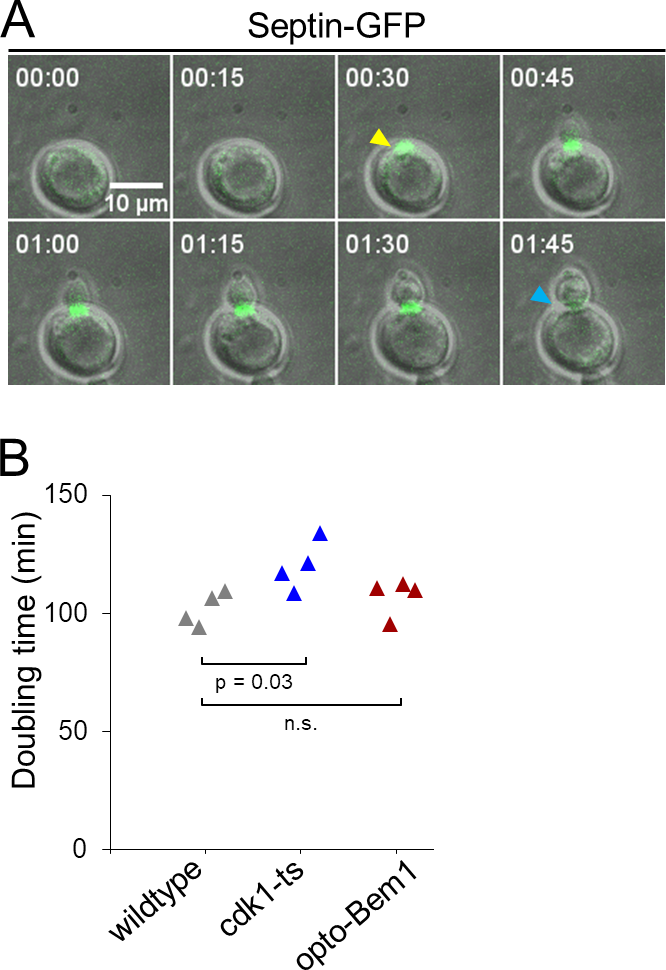

Supplement: S3 Fig — (A) opto-Bem1 cells were illuminated for 6–8 h with red light (to generate giant yeast), then switched to IR light (allowing giant yeast to bud and divide). Similarly, cdk1-ts cells were incubated at 37°C for 8 h (to generate giant yeast), then shifted to 25°C (allowing giant yeast to bud and divide). All cells were imaged every 5–10 min for ~8 h. Exogenously-expressed Cdc10-GFP was used to mark septin rings (green) and measure cell cycle progression. Panels depict representative opto-Bem1 cells. Budding duration, difference between the time of division (e.g., septin ring disappearance at ‘01:45’) and time of birth (e.g., septin ring appearance at ‘00:30’). ‘Mother volume’ was measured at the time of daughter cell birth (e.g., yellow arrow) and ‘daughter volume’ (i.e. only the former bud compartment) was measured at cytokinesis (e.g., blue arrow). Time, HH:MM. (B) Doubling times of indicated strains in liquid culture at 25°C during log-phase growth. (TIF) [file pone.0209301.s003.TIF]
