# Supplementary material for: A size-invariant bud-duration timer enables robustness in yeast cell size control
Source: PLoS One. 2018 Dec 21;13(12):e0209301. doi: 10.1371/journal.pone.0209301 (PMC6303054; doi:10.1371/journal.pone.0209301)
Supplement: S1 Supporting Information — (PDF) [file pone.0209301.s004.pdf]

Supplemental Experimental Procedures

1 Different models of growth

1.1 Growth proportional to surface area

We found that the radius r grows linearly with time t:

$$r(t) = at \tag{1}$$

where a is an arbitrary constant. It follows that

$$\frac{dr}{dt} = a \tag{2}$$

Ultimately, we are interested in how the volume changes over time ($\frac{dV}{dt}$) to be able to compare it with known growth models. We can calculate $\frac{dV}{dt}$ with the help of the known $\frac{dr}{dt}$ by:

$$\frac{dV}{dt} = \frac{dr}{dt}\frac{dV}{dr} \tag{3}$$

The volume and the radius are related by the formula for the volume of a sphere $V = \frac{4}{3}\pi r^3$. Calculating the derivative of the volume with respect to the radius

$$\frac{dV}{dr} = 4\pi r^2 = A \tag{4}$$

results in the expression for the surface area A of a sphere. Using equations (2) and (4) in (3) leads to

$$\frac{dV}{dt} = aA \tag{5}$$

This shows that if the radius grows linearly with time, the volume always grows proportionally to the surface area. Using the formulas for the surface area and the volume of a sphere, we see that plotting surface area over time would result in a quadratic curve, while the volume depends on time to the power of 3.

1.2 Growth proportional to volume

For comparison, we briefly describe what growth proportional to the volume would look like. Growth proportional to volume means that

$$\frac{dV}{dt} = aV \tag{6}$$

Solving this leads to

$$logV = at + C \tag{7}$$

where C is an arbitrary constant. Using the formula for the volume of a sphere, we can express the volume as a function of the radius:

$$log\left(\frac{4}{3}\pi r^3\right) = at + C \tag{8}$$

This can be rewritten as

$$\frac{4}{3}\pi r^3 = e^{at+C} \tag{9}$$

Solving for r leads to

$$r(t) = \left(\frac{3}{4\pi}\right)^{\frac{1}{3}} e^{\frac{1}{3}(at+C)} \tag{10}$$

This means that r would grow exponentially with time. The same is true for the surface area A and the volume (by using the expression for r in equation (10) in $A = 4\pi r^2$, $V = \frac{4}{3}\pi r^3$).

1.3 Constant growth

Another common model of growth is constant growth with respect to the volume, where

$$\frac{dV}{dt} = a \tag{11}$$

Solving this leads to

$$V = at + C \tag{12}$$

Including the volume as a function of radius

$$\frac{4}{3}\pi r^3 = at + C \tag{13}$$

leads to the following dependence of r on time t:

$$r(t) = \left(\frac{3(at+C)}{4\pi}\right)^{\frac{1}{3}} \tag{14}$$

This shows that in case of constant growth with respect to the volume, the radius would grow proportional to $t^{\frac{1}{3}}$, the surface area proportional to $t^{\frac{2}{3}}$, and the volume (as the name of this model says) proportional to t.
